# “As a woman who watches how my family is… I take the difficult decisions”: a qualitative study on integrated family planning and childhood immunisation services in five African countries

**DOI:** 10.1186/s12978-021-01091-1

**Published:** 2021-02-15

**Authors:** Jenna Hoyt, Shari Krishnaratne, Jessie K. Hamon, Lydia Boudarene, Tracey Chantler, Shiferaw Dechasa Demissie, Justine Landegger, Easterlina Moseti, Seth Marcus, Misozi Kambanje, Shannon Pryor, Nathaly Spilotros, Marius Gnintoungbe, Dora Curry, Jayne Webster

**Affiliations:** 1 Department of Disease Control, Faculty of Infectious and Tropical Diseases, London School of Hygiene & Tropical Medicine, London, UK; 2 Department of Global Health and Development, Faculty of Public Health and Policy, London School of Hygiene & Tropical Medicine, London, UK; 3 International Rescue Committee Ethiopia Program, Addis Ababa, Ethiopia; 4 International Rescue Committee US, New York, USA; 5 World Vision International, Nairobi, Kenya; 6 World Vision US, Monrovia, CA USA; 7 Save the Children International, Blantyre, Malawi; 8 Save the Children US, Washington, DC USA; 9 Care Benin, Cotonou, Benin; 10 Care USA, Atlanta, USA

**Keywords:** Family planning, Contraceptives, Women, Decision making, Integration, Childhood immunisations, Sexual and reproductive health

## Abstract

**Background:**

Family planning (FP) has the potential to improve maternal and child health outcomes and to reduce poverty in sub-Saharan Africa. However, substantial unmet need for modern contraceptive methods (MCMs) persists in this region. Current literature highlights multi-level barriers, including socio-cultural norms that discourage the use of MCMs. This paper explores women’s choices and decision-making around MCM use and examines whether integrating FP services with childhood immunisations influenced women’s perceptions of, and decision to use, an MCM.

**Methods:**

94 semi-structured interviews and 21 focus group discussions with women, health providers, and community members (N = 253) were conducted in health facilities and outreach clinics where an intervention was delivering integrated FP and childhood immunisation services in Benin, Ethiopia, Kenya, Malawi and Uganda. Data were coded using Nvivo software and an analytical framework was developed to support interpretative and thematic analyses on women’s decision-making about MCM use.

**Results:**

Most women shared the reproductive desire to space or limit births because of the perceived benefits of improved health and welfare for themselves and for their children, including the economic advantages. For some, choices about MCM use were restricted because of wider societal influences. Women’s decision to use MCMs was driven by their reproductive desires, but for some that was stymied by fears of side effects, community stigma, and disapproving husbands, which led to clandestine MCM use. Health providers acknowledged that women understood the benefits of using MCMs, but highlighted that the wider socio-cultural norms of their community often contributed to a reluctance to use them. Integration of FP and childhood immunisation services provided repeat opportunities for health providers to counter misinformation and it improved access to MCMs, including for women who needed to use them covertly.

**Conclusions:**

Some women chose to use MCMs without the approval of their husbands, and/or despite cultural norms, because of the perceived health and economic benefits for themselves and for their families, and because they lived with the consequences of short birth intervals and large families. Integrated FP and childhood immunisation services expanded women’s choices about MCM use and created opportunities for women to make decisions autonomously.

## Plain English summary

The use of family planning (FP) to space or limit births can improve the health of women and their children and can help alleviate poverty in sub-Saharan Africa. Women face a number of difficulties when deciding whether to use a modern contraceptive method (MCM) as it is commonly not accepted by their communities. This study sought to understand how women make choices about using MCMs and the forces influencing their decision to use them. It also looked at whether providing FP alongside childhood immunisation services would help women access MCMs.

A total of 253 women, health providers and community members participated in interviews and focus group discussions in Benin, Ethiopia, Kenya, Malawi and Uganda at sites where FP services and childhood immunisations were integrated. Nvivo software was used to code themes and aid in analysis.

Women felt that using MCMs helped them to delay or space births, which improved their health and the health of their children. They also had more time to work and manage their families. However, they feared the potential side effects of MCMs and were influenced by misinformation about MCMs. Husbands often disapproved of women using MCMs and there were reports of women using MCMs without their husband’s knowledge. Health providers agreed that women understood the benefits of using MCMs but that side effects and unsupportive husbands made some women reluctant to use them. By integrating FP and childhood immunisation services, women were given correct information that helped de-bunk myths about MCMs causing infertility and other health issues. It also helped women access MCMs without having to say they were attending FP services.

## Background

Family planning (FP) has the ability to reduce maternal and infant deaths and alleviate poverty by helping women to space and limit births [1]. The benefits of FP for women extend beyond improved health; declining fertility is associated with increased earnings and participation in employment for women [2]. However, a large unmet need for modern contraceptive methods (MCMs) persists in sub-Saharan Africa (SSA). This hinders progress towards the third and fifth Sustainable Development Goals, which call for action to improve health and well-being and achieve gender equality [3]. In low and middle income countries, 218 million women of reproductive age want to avoid pregnancy but are not using MCMs [4]. Given the renewed international recognition of sexual and reproductive health as a human right and the commitment to eliminate the unmet need for FP made at a the Nairobi Summit in 2019 [5], there is a clear need for a comprehensive understanding of factors that influence women’s decision-making about MCM use.

A woman’s desire to delay or stop childbearing is separate from her decision to act on that preference. Evidence indicates that a woman’s own desire to use an MCM is not sufficient to ensure uptake [6]. There are factors, external to a woman’s personal reproductive preference, that influence decision-making on MCM use. For many women, societal and community values, beliefs, and traditions, much of which are under pinned by gender-power dynamics [7], blur the decision-making space. Several studies have highlighted the influence of myths and misconceptions on MCM use. Fears linked to side effects, infertility and unsubstantiated health concerns have been explored in Rwanda [8], Kenya [9], and Uganda [10] and were mentioned by women as major reasons for non-use of MCMs. The Kenyan study noted that women (self-reported MCM users and non-users) learned about the side effects of MCMs from their peers, partners, and families, rarely mentioning health providers as a source of information. When it comes to FP, many women lack decision-making power regarding their family size and the use of MCMs [11]. Clandestine contraceptive use, defined as a woman’s use of a contraceptive method without her husband’s knowledge, is estimated to account for 6–20% of all contraceptive use in SSA [12].

When women choose to act on their reproductive preferences, health system structures and resource availability can enable or constrain their decision to use an MCM. Barriers affect a woman’s decision to use an MCM by limiting access, including: distance to FP services, service restrictions, and the availability and range of methods on offer [13–15]. Health provider attitudes and quality of FP services also influence the uptake of MCMs [1, 14]. Multiple efforts to extend FP services by integrating these into other reproductive and sexual health and child immunisation programmes have been documented. For instance, MCM use was associated with completion of the third dose of the diphtheria-tetanus-pertussis (DPT3) immunisation among post-partum women in Ethiopia and Malawi [16]. However, there is a need to understand *how* integration can provide opportunities for women to sidestep external factors and act on their reproductive desires.

As the rate of contraceptive use in SSA lags far behind the global average [5] and given the imperative to end unmet need for FP, a better understanding of why women choose to use an MCM and how they act on that choice is needed. As part of a wider evaluation on a set of interventions integrating FP with childhood immunisation services, this paper explores the factors that influence women’s choices and decision-making about MCM use, from the perspectives of women, health providers and community members at sites across five countries in SSA. In addition, it examines how the integration of FP services with childhood immunisations influences both women’s reproductive choices and decision-making.

## Methods

### Intervention and study sites

The intervention was implemented in predominantly rural communities at health facilities and outreach clinics by non-governmental organisations (NGOs) between January 2015 and January 2018 (Table 1). The scale of the interventions ranged from implementing integrated services in 14 health centres in one region in Uganda to 114 health posts across two districts in Ethiopia. The integration model varied by country and site; but broadly the intervention had similar components and objectives. They all sought to improve access to and uptake of FP services by co-locating, to varying degrees, messaging, counselling and the provision of MCMs with childhood immunisations. In this study, co-location is taken to mean that women could access both childhood immunisations and MCMs during the same health visit, however, these two services were often administered by different health providers and/or at different points in time during that visit. MCMs included condoms, oral contraceptive pill, injectables, implants and the intra-uterine device. However, the availability of these methods varied by site and country. In general, the intervention components in each country included: health provider training on FP counselling and MCM administration; raising awareness in communities about FP through existing structures (including community and religious leaders, and peer influencers, such as expert clients or male champions); supplying a range of short- and/or long-acting MCMs; and, supporting ongoing provision of routine childhood immunisations.

**Table 1:** Integrated family planning and childhood immunisation interventions by country

Country	Implementing NGO	Scale of the intervention	Prevalence of modern contraceptive use in currently married women 15–49 years %	Proportion (%) of women who made FP decision jointly with husband/made FP decision on her own	Actors involved in the delivery of FP messages and services/delivery sites
Benin	CARE	*1 health zone:* Adjohoun-Bonou-Dangbo (ABD) health zone 19 health centres; 1 hospital	National: 12.4 Ouémé department: 15.2 [17]	*Oueme department:* Among FP users: 35/57.2 Among non FP users: 19.2/75.7	Services delivered at health facilities Midwives and nurses give FP counselling and administer MCMs Peer influencers mobilise women in the community to attend FP and immunisation services
Ethiopia	IRC	*2 districts:* Bambasi and Assosa districts, Benishangul Gumuz Regional State (BGRS) 114 health posts	National: 35.3 BGRS region: 28.4 [18]	*Benishangul-Gumuz:* Among FP users: 75.9/14.4 Among non FP users: 55.1/22.3	Services delivered at health posts and during 45-day post-partum home visits Community health workers (HEWs) give FP counselling and administer MCMs (except implant removals) Peer influencers (HDA) act as role models to encourage women to use MCMs, dispel harmful myths
Kenya	World Vision	*2 districts:* Garba Tulla, Isiolo county and Pokot West/Pokot South, West Pokot county 19 health facilities	National: 53.2 Isiolo: 26.3 West Pokot: 14.2 [19]	Data not available	Services delivered at health facilities Nurses provide FP counselling and administer methods Community health workers (CHVs) deliver health messages and mobilise women in the community to attend FP services Peer influencers (male champions) act as role models and share the benefits of using MCMs to space children
Malawi	Save the Children	*3 districts:* Blantyre, Mwanza, Thyolo 24 outreach clinics	National: 58.1 Southern region: 54.4 [20]	*Southern region:* Among FP users: 75.3/15.7 Among non FP users: 50.5/36.1	Services delivered at routine monthly outreach clinics Community health workers (HSAs) provide FP counselling and short-acting methods; nurses provide long-term methods but are not always present
Uganda	IRC	*1 region:* Karamoja 14 health centres	National: 34.8 Karamoja: 6.5 [21]	*Karamoja region:* Among FP users: 71.2/27.7 Among non FP users: 59.6/28.8	Services delivered at health facilities Midwives provide FP counselling and administer methods Community health workers (VHTs) deliver messages about FP and mobilise women to attend FP services using referral cards Peer influencers act as role models to encourage women to use MCMs, dispel harmful myths

*IRC* International Rescue Committee, *HEWs* Health Extension Workers, *HDA* Health Development Army, *CHVs* Community Health Volunteers, *HSAs* Health Surveillance Assistants, *VHTs* Volunteer Health Team

Regional data from the Demographic and Health Surverys (DHS) of countries included in this study indicates that among married women who are currently using FP, a high proportion of women reported being involved in decision making about FP, either jointly with their husband or making the decision themselves (range from 90.3 to 98.9%). However, among women who reported not using FP, women’s involvement with decision-making was lower (range from 77.4 to 94.9%)—data on decision making was not asked in latest DHS for Kenya. Unmet need for FP, defined as women who want to space or limit births but are not currently using FP, among married women ranged from 12.4% in the Eastern province in Kenya to 33.7% in the Oueme region of Benin [17–21].

### Data collection

Purposive sampling was used to select key stakeholders involved, or with an interest, in the intervention including implementing NGOs, health administrators, health providers (community- and facility-based), peer influencers, religious leaders, male community members, and women who self-reported as MCM users and non-users. Participants were selected through a consultative process with the implementing NGO in each country. Using this process, key stakeholders were identified based on an initial programme theory of how the intervention works [22] followed by maximum variation sampling amongst identified categories of stakeholders [23]. Providers were selected based on having experience in delivering either immunisation or FP services in health facilities where the intervention was perceived to have been more, or less, well received based on monitoring data collected by the implementers. They were approached in the study setting, at either health facilities or outreach clinics where the intervention was implemented, and asked to participate in the study. Interviews were conducted on site and were visible to others but out of earshot.

In total, 94 SSIs and 21 FGDs with 253 participants were conducted between October 2017 and March 2018. SSIs were used, when possible, because of the sensitive nature of the topics being discussed and to enable the interviewer to explore themes and gain individual perspectives in greater depth. FGDs were used to explore collective views and were conducted as part of the evaluation, when feasible, to understand where different groups of stakeholders might have similar or divergent views regarding aspects of FP. For instance, FGDs were sought with male community members to generate a rich discussion around the wider socio-cultural factors that influence perceptions about FP generally, and, MCM use specifically. Using a mix of SSIs and FDGs with women participants enabled both a deeper understanding of women’s individual perceptions towards FP and MCM use and opportunities to understand how perceptions about socio-cultural norms and FP practices may differ or not. Data from SSIs assisted the researchers in recognising if and when groupthink might be present in the FGDs [24].

Interview and discussion guides were developed for SSIs and FGDs, which were informed by local implementers. Questions were standardised across sites and countries to enable uniformity in the analysis framework, however, where specific contextual elements arose, interviewers were trained to explore those threads in greater detail. For both SSIs and FGDs, topics discussed with health providers included: workload, socio-cultural norms, healthcare access, delivery of integrated services and perceptions of women’s use or non-use of MCMs. For women, topics included: reasons for use or non-use of MCMs; barriers to MCM use; access to FP services; and, community-level acceptance of MCM use. And for community members topics included: socio-cultural norms, acceptance of FP, and perceptions of the integrated delivery of FP and immunisations. Interviews and discussions were conducted in each country by SK and a local researcher who was a trained interviewer and could facilitate a deeper understanding of the contextual factors that arose during the interviews and discussions. In Benin interviews were conducted in French and Ouémé; in Ethiopia in Amharic and English; in Kenya in Borana, Pokot and English; in Malawi in Chichewa and English; and in Uganda in Karamojong and English. All interviews were audio recorded, transcribed *verbatim* and then translated into English by experienced transcribers and translators.

### Analytical framework

To guide analysis an analytical framework was developed based upon the Sexual and Reproductive Health Empowerment framework by Karp et al. [25], which illustrates a woman’s empowerment journey across three phases: (1) *existence of choice*—where women have the capacity to recognise and set their reproductive goals, and how contraceptive use aids in achieving their reproductive goals, (2) *exercise of choice*—where women make decisions to act on those reproductive goals, and (3) *achievement of choice*—when women act to achieve their desired reproductive outcomes. Karp’s framework is useful because it acknowledges that reproductive desires are separate and distinct from the decision to use an MCM, which enables a deeper examination of the factors influencing women’s reproductive desires and their decisions to use an MCM to achieve their goals.

**Fig. 1:**
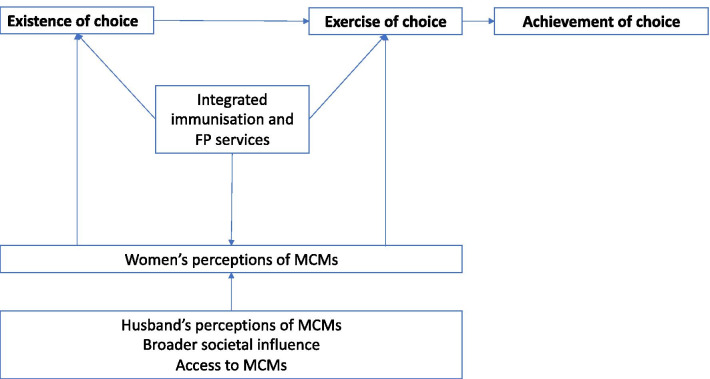
Analytical framework

In this paper, women’s decision-making about MCM use is explored within the context of integrated FP and childhood immunisation services. Our analytical framework (Fig. 1) builds upon the Karp framework for this purpose suggesting that women’s *existence of choice* (reproductive preferences/desires) and *exercise of choice* (decision to act on those desires) are influenced by women’s perceptions of MCMs and by external influences—such as a husband’s perceptions of MCMs, the socio-cultural context and access to MCMs. And further, it suggests that integrated FP and immunisation services may influence women’s reproductive desires and their decision-making about MCMs.

### Data analysis

The translated transcripts from the SSIs and FGDs were imported into NVivo 11.2 for coding and analysis. Transcripts were anonymized but the type of stakeholder attributable to each quote was retained to aid analyses. The data were coded and the primary analysis was conducted by JH and then discussed amongst the evaluation team to ensure a consensus was reached where ideas and opinions differed. It was agreed among the research team that data saturation was reached once no additional themes or sub-themes were being generated from the data [24]. The primary analyses were country specific with one coding framework used across all countries. The data were initially coded based on the major themes from the interview topic guides and included: (1) the actors involved in delivery and uptake of FP services; (2) the cultural and social context; (3) the delivery of the intervention; (4) decision-making of health providers and women; and (5) outcomes relating to the uptake of FP services and the use of MCMs. An iterative process was used, and additional themes and patterns were identified [26]. Interpretative syntheses were conducted to explore overarching themes across all five countries [27] including a thematic analysis that involved mapping themes to the analytical framework to identify: (1) internal motivations for MCM use and (2) external forces influencing the decision to act on the reproductive desires and (3) the role of integrated services in shaping women’s choices about MCMs and their decision-making on use. The Standards for Reporting Qualitative Research guidelines were used to ensure rigorous reporting of the study [28].

## Results

Across the five countries, a total of 72 women (self-reported MCM users and non-users), 83 health providers delivering FP and childhood immunisation services, 33 health administrators and implementers, and 40 community members (religious leaders and peer influencers) were interviewed (Table 2). Additionally, in Benin and Kenya, 25 male community members were interviewed. These categories represent the primary role for which the participant was interviewed. However, among the religious and community leaders, and peer influencers, there were men who shared their views on MCM use within their family and women who discussed their personal use of MCMs. Women, MCM users and non-users, were of reproductive age and from predominantly rural communities.

**Table 2:** SSIs and FGDs by participant groups

	Women: MCM users & non-users	Health proivders: facility/community based	Health administrators/implementers	Community members: religious leaders, peer influencers*, community leaders	Male community members
Benin	3 FGDs (20)	6 SSIs	4 SSIs	None	2 FGDs (10)
Ethiopia	1 SSI	7 SSIs	6 SSIs	9 SSIs	None
Kenya	4 FGDs (43)	5 SSIs & 3 FGDs (23)	5 SSIs	2 SSIs & 2 FGDs (20)	2 FGDs (15)
Malawi	7 SSIs	3 SSIs & 4 FGDs (25)	10 SSIs & 1 FGD (3)	2 SSIs	None
Uganda	1 SSIs	14 SSIs	5 SSIs	7 SSIs	None
Total participants	72	83	33	40	25

() indicates number of participants in the FGDs

*These include women who were MCM users

### Existence of choice

The *existence of choice* reflected women’s reproductive preferences, which were found to be driven by their personal desire to space or limit births, and shaped by their lived experience. Findings also revealed how the *existence of choice* encompassed women’s perspectives about contraceptive use, which was influenced by their positive and negative perceptions of MCMs and how these aligned with their reproductive preferences.

#### Women’s reproductive preferences: internal motivations and lived experience

At all sites, women expressed a desire to space their children, primarily because of the perceived health benefits for themselves and their children, including the ability to feed and care for their children. This preference for healthy birth spacing was rooted in the daily hardships described by women across all contexts. In Uganda, food insecurity was commonly mentioned by women who wanted to space births in order to ensure their children had enough to eat. However, food insecurity was also mentioned in Malawi and Kenya as influencing reproductive preferences. Negative health effects on both mother and children, linked to inadequate spacing, were frequently described by women at all sites, and the lack of spacing and large family size was also perceived to impede a child’s prospect for growth and future opportunity.

*“Respondent (R): Family planning is good because you can monitor your child then you can know that you can manage to feed that one or even I am done with even two children let me first feed these ones or even paying* [school] *fees until they finish…”* Peer influencer and MCM user 21, Uganda

*“R: I saw that it’d help me because it is us women who struggle with childbearing. We lose a lot of blood during childbirth so during child spacing, we have the opportunity to regain the lost blood so that why I decided to go for family planning.”* Woman MCM user 14, Malawi

Women perceived healthy spacing to have a positive effect on the welfare of the entire family. These benefits included enabling women to have time to work and improve the economic situation of their family. Whilst for some women, particularly at sites in Benin and Uganda, using MCMs was a way to reduce domestic tensions, as they no longer needed to refuse sex with their partner, for fear of becoming pregnant.

*“R: Family planning brings harmony to a household and can mean that even the woman is free to pursue her work for the benefit of the entire household.”* Women FGD (MCM user) 5, Benin

Importantly, the reproductive preference, to space or limit births, reported by women in this study was largely motivated by their own internal desire to both preserve their health and enable their children to thrive, which often differed from their husbands’ preferences. However, this internal desire was inextricably linked to the context of rural poverty described by participants. Women, health providers and community members acknowledged that economic challenges—including the impact of food insecurity—were driving changes to social norms about family size, which was reflected in women’s fertility preferences.

*“R: let me begin from those who choose, what it depends much is our land is arid and one choose to do spacing because of famine since when you are few and the food is little, it can be enough until when you find another one. And that is why they decide it is better to space even if they stay for three years before getting another one the situation may have change.”* Community health volunteer 16, Kenya

*“Interviewer [I]: So what does your husband think about family planning? R: He thinks but he doesn’t think, he thinks about producing only but my heart thinks about family planning.”* Woman MCM non-user 23, Uganda

#### Perceptions of MCMs: the balance between useful and harmful

Women’s *existence of choice* was shaped by their perceptions of MCMs. For some women, MCMs were perceived as a useful tool to help them space or limit births. Women who had been successful at spacing births naturally did not perceive a need for MCMs to achieve their reproductive goals. For women who preferred to limit childbearing, MCMs were recognised as a way of achieving their goal.

*“I have never been on any contraception in the past and that was my choice. I have 5 children who all have between 2 to 3 years between them. I have spaced them out naturally without using contraception and I preferred that. Now that I’ve had my 5th and final child; I don’t want more children, I have planned to come to the health post and use the family planning services.”* Woman non-MCM user 9, Ethiopia

However, participants at all sites described myths and misconceptions about MCMs that influenced women’s perceptions and limited women’s *existence of choice*. Internalised fears about MCMs leading to infertility and causing illness were reported across all countries. These fears tapped into important social and gender norms that place value on a woman’s ability to bear children. For some women, particularly in Ethiopia and Kenya, religious beliefs underpinned their perceptions about MCM use and constrained their *existence of choice.*

*“R: like implant, women do complain a lot about that they have excess bleeding, others do miss even their menses and like last time there was [a woman] who had to go under surgery… for the implant to be removed, so we fear because of all this and if all can be cleared at once that they are misconception or even true, doctors need to clarify for us… because with all those rumors no one is ready to die, we rather be giving birth each year.”* Women FGD (MCM non-users) 6, Kenya

Exposure to correct information about FP and MCMs was found to help women and the wider community understand the benefits of using MCMs, and helped to counter harmful misinformation, which in turn had shaped perceptions of MCMs. In both Kenya and Ethiopia, religious leader acceptance of MCM use was perceived to expand opportunities for women to make choices about MCM use.

*“R: They might say that if you die and the implant is still inside you then it is against the Muslim religion, as you are not supposed to be buried with any foreign object inside you. They also say preventing a baby is Haram* [forbidden]. *Together with some religious leaders, we are in the process of changing people’s perceptions about family planning.”* Nurse 23, Ethiopia

### Exercise of choice

The *exercise of choice* reflected women’s decision-making about MCM use, which was guided by their desire to space or limit births and catalysed by their recognition that they suffer the consequences of unspaced, frequent births and large family size. External to their personal desire for MCM use, women’s decision-making was strongly influenced by fear and experience of side effects, unsupportive husbands, and their peers.

#### Reproductive preferences guide women’s decision-making on use of MCMs

Women’s decisions to act and take up an MCM was strongly influenced by their desire to space births and capitalise on the perceived health and wellbeing benefits for themselves and their children. Women at all sites acknowledged that it was them who struggled, as men did not feel the negative consequences of frequent childbirth and of caring for multiple young children, and that was often described as a catalyst for taking up an MCM.

*“R: We want to space our children, who wants to give birth every year and you know men are not that much concerned if it comes to pregnancy, so you decide to take extra care of yourself…”* Woman MCM user 7, Kenya

*“It means you can have as many children as you want, when you want them. You might even want ten or twenty children and family planning allows you to have as many children as you want, but more importantly when you want them and in a way that ensures all your children can thrive. It means you won’t have several young children and be pregnant at the same time.”* Women FGD 7, Benin

Health providers also noted the importance of women’s personal reproductive desires and used messages that highlighted the consequences to motivate women’s decision-making about MCM use.

*“R: So, that is why we tell the mother, it is you who makes the decision. Because now, your husband can say no, but it is you who carries that burden. When you tell about these real facts, they can see yeah it is true, that the women… every year she produces… At least now, now I have hope. This family planning is good…”* Midwife 8, Uganda

#### External forces influencing the decision to use MCMs

Women described factors external to their own reproductive desires that shaped their decision to use or not use an MCM. These included side effects, husbands, and peer influence. Some external forces, such as husband disapproval of MCM use, were found to influence women’s decision making without altering their positive perceptions of MCMs. Other forces, such as side effects forced women to weigh the benefits and risks associated with MCM use.

Fears of side effects were widely reported and generally contributed to a reluctance to use MCMs. These fears were reported by women who experienced side effects themselves and by women who heard about side effects from others. At several sites, this fear was linked to the idea or experience of increased blood loss and irregular bleeding. The loss of blood had different contextual meanings and was embedded in the social norms of the community. For instance, women in Ethiopia feared blood loss outside their normal menstruation. In Kenya, irregular spotting resulted in disruptions to domestic tasks, due to the belief that women are unclean when they bleed, and increased domestic tensions.

*“R: I was afraid about [the] implant…because some people explain more about this thing… Especially the negatives…They say they don’t have appetite… for food, even for sex; they say it is troublesome because they develop other diseases… like headaches and stomach pains, like those things. Menstruations, it’s a problem; continuous menstruations…”* Woman MCM user 13, Malawi

Women in all countries reported that unsupportive husbands posed a barrier to MCM use. Many women recognised that child rearing and spacing was an issue that mainly affected them but acknowledged that they often needed their husband’s approval to use MCMs. Women reported various reasons for the lack of approval among husbands in their communities, such as discordant fertility preferences and fears about MCMs causing infertility. However, gender dynamics were also a factor. Across all sites, but most prominently in Benin, husbands’ disapproval of MCM use was linked to fears that it encouraged female promiscuity and prostitution. In Malawi, women believed husbands’ resistance to MCMs was fuelled by fears of MCMs disrupting sexual relations.

*“R: My older brothers don’t like it. In their opinion, the family planning corrupts the mind of women in couples. They no longer respect their husbands.”* Men’s FGD, 15, Benin

Women described situations where they used MCMs without their husband’s permission. Some women acknowledged it would cause domestic tensions if their husband found out but they were resolute in their justification for doing so because they believed men didn’t consider the consequences of frequent childbirth.

*“R: he [husband] doesn’t want [FP]… but for me as a woman who watches how my family is because he knows nothing about his family and that is why I take the difficult decisions and I also go without letting him know … he will not accept, my responsibility is also to practice without his knowledge.”* Peer influencer and MCM user 18, Uganda

Conversely, some women reported having supportive husbands who accepted their use of MCMs as a means of birth spacing, although this was less prominent in Benin. At all sites, a lack of resources, including food and money, was reported as the main reason for husband’s support. Some husbands also accepted the use of MCMs given the health concerns related to frequent childbirth. Joint decision-making about MCM use was most often described by women at sites in Malawi.

*“I: Why does your husband support family planning? R: Because of school fees and hunger….One can marry three wives with each of them having either five, others four and so not all houses will be able to provide for her family.”* Woman MCM user 20, Kenya

Women reported hearing stories, experiences and rumours from friends, peers and the wider community about the use of MCMs. This had positive and negative effects on their desire to use MCMs. Stories and experiences about side effects, complications, and infertility were all reported to have negatively influenced women’s adoption of MCMs. However, several women reported being motivated to take up an MCM by talking to a friend or hearing about the positive effect that FP had on a woman in their community. That is, witnessing the benefits MCM use had on others was seen to further galvanise women to pursue their own reproductive goals.

*“R: I’ve got an older sister who got the implant, and everyone told her about the negative side effects. People talked about them so much that she offered to go and get it removed and when she got it removed, she never had more children*.” Woman MCM non-user 5, Benin

*“R: So when I saw the advantages of family planning from my friends who are accessing. I saw it could help me in my life. Immediately, I started to see the goodness of family planning.”* Woman MCM user 11, Malawi

Some women, who reported not using an MCM despite a desire to space or limit births, revealed that their decision-making was dominated by external forces.

*“R:…I think FP is a good thing because it means we won't give birth again too soon after the last one. But I’d like to add something. In our tradition, if a woman wants to do something, she has to tell her husband…”* Women’s FGD (MCM non-user) 5, Benin

Access to MCMs also influenced women’s decision making. In particular, access to discreet methods enabled women to make autonomous decisions about MCM use. Women’s preference for discreet methods was emphasised in Malawi—where women frequently opted for injectables—and this was perceived by health providers to be, in part, due to the ease with which women could use it covertly.

*“R: So they prefer injectable drugs…for many women who are taking Depo-Provera they say it’s more secretive, even to their husbands or to other women…”* Community nurse 9, Malawi

#### The role of integration: expanding choice and improving access to MCMs

The integration of services was found to expand the *existence of choice* by repeatedly exposing women to correct information about the use of MCMs to space or limit births. The presence of harmful misinformation about MCM use (e.g. that it causes infertility or other illnesses) was reported by many respondents across all sites and was cited as a reason for non-use of MCMs.

*“R: We had a lot of gaps in the community’s understanding of what contraceptives could do. Since the integration, the mothers get advised when they come in to get their child vaccinated. Using the immunisation as an opportunity to increase their understanding of the pros and cons has really worked.”* Health provider 5, Ethiopia

The integration of FP messages during childhood immunisations provided women with information about the benefits of FP and MCM use at a time when they were receptive to hearing about health benefits for themselves and for their child. These messages resonated with women and aligned with their reproductive desires and, for some women, catalysed their decision to act.

*“R:…I think that it is great that they use immunisation as an opportunity to talk to us about FP. I say it’s a good thing because when you go to the health centre to immunise your children, it’s for their well-being. So, when health workers talk to us about how FP can benefit us, our children and our family, and we choose to do this….”* Women FGD participant (MCM user) 7, Benin

The co-location of services was perceived to have increased opportunities for women to act on their reproductive desires by improving access to MCMs (including discreet methods) and enabling women to sidestep seeking permission from their husbands. For example, in Malawi, the integration of FP services in outreach clinics brought services closer to women in remote communities. Similarly, in Ethiopia, the use of trained Health Extension Workers to deliver FP services enabled women to access these services at local health posts and during 45-day post-partum home visits.

*“R: first, no time wasted because you take child for immunisation and the doctors tells you about family planning, the rest depend with your decision…*” Woman MCM user 7, Kenya

Crucially, for women requiring covert MCM use, the integration of FP with childhood immunisations provided an alternative reason for women to attend health facilities whilst gaining access to FP services. However, in the Benin context where community stigma around MCM use was high, this co-location of services enhanced the visibility of MCM use, resulting in women asking providers to collect their MCM at night-time to avoid being seen.

*“R: While us women are the ones who see the problem and therefore go to access. Men cannot force us not to access family planning. I: What if a man refuses? What can you do? R: I can come and buy a health passport and leave it with the doctor, then start family planning.”* Woman MCM user 11, Malawi

## Discussion

This study found that women were often compelled to act on their reproductive desires and adopt an MCM despite having to overcome significant external forces, such as the fear of side effects and disapproving husbands. Women’s desire to space or limit births reflected their own internal preference, based on their lived experiences. However, their perceptions of MCMs were found to be influenced by powerful misconceptions about infertility and illness. The integration of FP with childhood immunisation services influenced women’s perception of MCMs through increased opportunities for health providers to counter harmful misconceptions about MCMs. It also influenced women’s decision-making by improving access to MCMs and, through the ability to circumvent unsupportive husbands, to act on their reproductive preferences. For health providers, this deeper understanding of what factors influence women’s *existence* and *exercise of choice* could inform the development and delivery of FP messaging. That is, by understanding women’s personal reproductive preferences health providers can then help women navigate the external forces obstructing their decision to act—through appropriate management of side effects, de-bunking infertility myths, and supporting covert contraceptive use.

Women’s reproductive aspirations transcended geography, cultural contexts and reported MCM use. Findings suggest that women’s desire to space or limit births was driven by their lived experience of hardship and struggle. This reflected the daily challenges of poverty and the physical toll of frequent pregnancies, but was exacerbated by gender dynamics that put the burden of childcare largely on women. This internal desire to space or limit births could be interpreted as women simply internalising gender inequality but it is important to acknowledge a small but key distinction: women setting their reproductive goals based on their own lived experience rather then a husband’s fertility preferences or social norms favouring large families—is the *existence of choice*. That is to say, the woman’s own lived experience has shaped her reproductive choice. However, women’s choices about MCM use were found to reflect wider societal influcences.Women’s perceptions of MCMs were often embedded in religious beliefs or represented internalised fears about infertility and illness linked to MCM use. These findings are echoed by Karp [25], who noted that fears about infertility often constrain women’s choices about MCM use. In this study, hearing negative stories about MCMs from others was frequently reported by participants and was linked to non-use. This relationship between a woman’s belief in myths about FP and the non-use of MCMs is consistent with other studies [9, 10, 29, 30]. Findings from this study suggest that integrating FP messaging into childhood immunisations can offer repeated opportunities for women to hear a counter-narrative to the rumours circulating. In addition, integrated messaging gives women a chance to learn how MCMs could help them achieve their reproductive goals at a time when they are receptive to receiving this information. A study looking at missed opportunities for integrating FP services for postpartum women in Ethiopia and Malawi found that contraceptive uptake was more likely when FP services were integrated with immunisations, when compared with antenatal care, and suggested that women are more receptive to information when given at a time when they can act on it [16].

This study demonstrated that a woman’s decision to use an MCM was driven by her reproductive preference to space or limit births, but strongly influenced by external forces. Side effects emerged as a powerful deterrent to MCM use by women in this study. This link is well established in the literature [31, 32]. DHS data from countries included in this study indicate discontinuation rates because of side effects (for all MCMs) range from 10 to 35%, which represents a significant obstacle to increasing MCM uptake [17–21]. Crucial for implementers and health providers is understanding why side effects cause women such distress. Counselling about potential side effects and how to manage these, either medically or by switching methods, is key to minimising the physical discomfort and pain associated with MCM use. In Karp’s framework, self-efficacy is a construct linked to the *exercise of choice*. A study linking mechanisms to acceptability constructs indicated that when women felt able to discuss side effects and their management with a health provider, it triggered feelings of self-efficacy, which was in turn linked to increased acceptability of MCMs [33]. However, there are contextual factors that need unpacking, such as the different cultural meanings associated with blood loss. For instance, in some contexts irregular bleeding from MCM use constrains a women’s ability to carry out daily tasks because of the belief that women are unclean when they bleed. However, these constraints on women’s movements reflect the unbalanced power dynamics between men and women that manifest in menstrual taboos and social control [34] and not the physical symptoms caused by MCMs.

Women’s decision-making about MCM use was also found to be influenced by disapproving husbands, a finding that mirros results from studies conducted in other countries in SSA [8, 14, 35]. However, the co-location of FP services with childhood immunisations provided an alternative reason for women with young children to attend the health facility or outreach clinic and, in doing so, facilitated access to FP services. This, coupled with the availability of concealable methods, enabled women to make FP decisions free from some of the external constraints that limited their decision-making power. Additionally, health providers were willing to provide MCMs to women without requiring a husband’s permission. A study in Kenya found that women reported service restrictions at the provider level, such as requiring their husband’s approval, as a reason for not continuing with a contraceptive method [14]. Integrated services that are flexible is important in order to respond effectively to contextual factors. In the context of Benin’s communities, the stigma associated with use of MCMs, linked to unsupportive husbands, meant that referral systems between the two services had to be altered so that women were not identified as MCM users. Additionally, health providers in this context made the decision to support women’s use of MCMs by allowing them to collect the methods at night and avoid being seen. Had providers opted not to facilitate women’s requests for discrete service delivery times, uptake of MCMs would have been negatively affected.

Many of the barriers to MCM use, including the cultural issues with blood loss and lack of support from husbands, are supported by patriarchal structures that reinforce gender norms conducive to male dominance over women [36, 37]. MCM use threatens this partriarchal hierarchy because women’s reproductive enslavement has long been used as a means of maintaining male dominance [38, 39]. Many women in this study remarked on how the burden of caring for children fell to them yet acknowledged that they were often not the primary decision-maker about family size. This finding is echoed by researchers who investigated contraceptive use in Rwanda [8] and noted that although FP was considered a woman’s matter, men often exerted decision-making power over MCM use. In our study, recognition of this imbalance was found to be a common catalyst for women to act on their reproductive preferences and use MCMs. Covert use is a way in which women try to right this imbalance and make autonomous decisions based on their reproductive preferences. Recent research suggests this practice is commonplace in other countries within SSA [40, 41]. How gender dynamics underpin women’s choices, reproductive desires and decision-making is key to designing effective FP services that acknowledge these constraints and support women’s autonomous decision-making. Key recommendations from this study are that: (1) health providers need training that addresses cultural and traditional gender norms and that promotes high quality services that supports a woman’s autonomy and right to use MCMs; (2) communication about the expectation and management of side effects is central to FP counselling; and (3) gender transformative male engagement that deepens men’s understanding of women’s reproductive preferences and promotes equal decision-making within couples is crucial.

By exploring the views of women, health providers and community members, this study provides broad perspectives on the factors influencing women’s decision-making about MCM use. However, this study is not without limitations. It is possible that social desirability bias was an issue among all participant groups, however, the range of viewpoints that emerged from within participant groups would suggest that it was not a major issue. For example, views among women ranged from disapproval of MCM use to open acknowledgment of personal covert MCM use. Respondent roles varied across study sites as the selection of participants used a purposive approach and was informed by an initial programme theory developed by programme designers and implementers [22]. For example, some of the women interviewed had two roles (both MCM user and peer influencer) and thus their perspectives might differ from the average MCM user. Additionally, participants in this study were selected from within integrated FP and childhood immunisation interventions and therefore the perspectives of women who do not attend these services may differ. A further limitation is that interviews were coded by a single researcher. The analysis was therefore inevitably shaped by the lens through which this researcher interpreted the data. To limit this effect, discussions with the research team were held and where disagreements arose concensus among the team was sought. Additionally, validation of the themes and findings was carried out with the implementation teams.

## Conclusion

Women use MCMs because of the health and economic benefits to themselves and to their families but also because they struggle with the realities of frequent births and large family size. Unsupportive husbands and MCM side effects were found to influence women’s decisions about MCM use without shifting their internal desire to space or limit births. Integration enabled repeat opportunities for women to hear messages about the benefits of MCM use, which helped counter misinformation and modified women’s perceptions about MCMs. In addition, integration was perceived to have improved access to MCMs and to have enabled some women to make autonomous decisions about using an MCM. Overall, implementers should be cautious and flexible to ensure the way in which co-located services are delivered does not enhance stigma. Understanding what underpins women’s reproductive choices and decisions about MCMs use can offer important insight for designing effective interventions that support women in overcoming barriers to achieve their reproductive goals.

## Data Availability

The datasets used and/or analysed during the current study are available from the corresponding author on reasonable request.

## Abbreviations

CHV: Community health volunteer
FGD: Focus group discussion
FP: Family planning
HEW: Health extension worker
HSA: Health surveillance assistant
I: Interviewer
MCM: Modern contraceptive method
NGO: Non-Governmental Organisation
R: Respondent
SSA: Sub Saharan Africa
SSI: Semi-structured interview
VHT: Volunteer health team
